# Husband’s involvement with mother’s awareness and knowledge of newborn danger signs in facility-based childbirth settings: a cross-sectional study from rural Bangladesh

**DOI:** 10.1186/s13104-018-3386-6

**Published:** 2018-05-09

**Authors:** Sojib Bin Zaman, Rajat Das Gupta, Gulam Muhammed Al Kibria, Naznin Hossain, Md. Mofijul Islam Bulbul, Dewan Md Emdadul Hoque

**Affiliations:** 1 Maternal and Child Health Division, International Centre for Diarrhoeal Disease Research, Bangladesh (icddr,b), Dhaka, Bangladesh; 2 James P Grant School of Public Health, BRAC University, Dhaka, Bangladesh; 3 Department of International Health, Johns Hopkins Bloomberg School of Public Health, Baltimore, USA; 4 Department of Pharmacology, Dhaka Medical College, Dhaka, Bangladesh; 5 Public Health and World Health Wing, Ministry of Health and Family Welfare, Dhaka, Bangladesh

**Keywords:** Husband involvement, Knowledge, Newborn danger sign, Bangladesh

## Abstract

**Objective:**

The aim of this study was to examine the association between husband involvement and maternal awareness and knowledge of newborn danger signs. This cross-sectional study was conducted in three rural hospitals of Bangladesh among the recently delivered women (RDW).

**Results:**

RDW were interviewed to determine their knowledge and understanding of seven key neonatal danger signs. About 51.4% of the respondents were able to identify at least one danger sign. ‘Fever’ was the most correctly identified (43.7%), and hypothermia was the least (26.1%) identified danger sign. The factors associated with RDW possessing knowledge of at least one neonatal danger sign were: secondary education (COR: 1.3, 95% CI 1.1–1.6), increased ANC visits (COR: 1.2, 95% CI 1.1–1.3), previous history of facility delivery (COR: 1.3, 95% CI 1.1–1.4), and husband involvement in the mother’s facility delivery (COR: 1.3, 95% CI 1.1–1.5). RDW were more likely to recall at least one newborn danger sign (AOR: 1.2, 95% CI 1.1–1.4) when the husband was actively involved in his wife’s antenatal, delivery and postnatal care. In conclusion, this study found that husband involvement was significantly associated with the maternal knowledge related to identification of neonatal danger signs.

**Electronic supplementary material:**

The online version of this article (10.1186/s13104-018-3386-6) contains supplementary material, which is available to authorized users.

## Introduction

Of the estimated 5.9 million under-five deaths in 2015, about 45% of children died in the neonatal period. The highest number of neonatal mortality occurred in South Asia with a neonatal mortality rate (NMR) of 30 per 1000 live births in 2015 [1]. Many countries in this region have a high NMR including Bangladesh with an estimated NMR of 28 per 1000 live births [2]. A child survival revolution has been observed due to the successful implementation of different interventions which have contributed to lower rates of neonatal mortality [3–5]. Previous studies found that timely care-seeking is essential during illness to achieve the targeted reduction of maternal and neonatal mortality [6, 7].

Earlier studies estimated that 80% of neonatal deaths are preventable with increased coverage of currently available, evidence-based, and cost-effective measures [8, 9]. In particular, early symptom recognition, appropriate care-seeking, and recognition of danger signs have been identified as cornerstones in neonatal death reduction [10, 11]. As neonates are more prone to express subtle signs of illness [12, 13], it is essential that mothers possess the knowledge needed to identify symptoms. Symptoms such as breathing difficulty, very low or high temperatures, convulsions, feeding problems and less movement are symptoms of the leading causes of neonatal deaths—notably neonatal sepsis, perinatal asphyxia, and prematurity [14].

In a traditional Bangladeshi family, the husband acts as the major decision maker, and the wife and children respect the decisions that are made [15]. Consistent with this family model, Mullany and Thapa found that it was essential to involve husbands when looking to improve the health of pregnant women and their newborns [16, 17]. Other studies that examined the factors associated with maternal ability to identify newborn danger signs weren’t successful at the facility level [18, 19]. The current study investigated the extent to which a mother can recognize the danger signs of newborn illness, and how husband involvement influenced the knowledge level of these women.

## Main text

### Design and methods

#### Study design, settings, and participants

This cross-sectional facility-based study was conducted from January to April 2015. The study was carried out in three Upazila (i.e. sub-district) Health Complex (UHC) facilities in the Tangail district of Bangladesh. Each sub-district contains a UHC which is the secondary level referral hospital with a 50 bed inpatient capacity for a catchment area with a population of 300,000–500,000. For this study, we enrolled 142 recently delivered women (RDW) who encountered a normal vaginal delivery (NVD), had a live born baby, and were physically stable. Each mother was accompanied by at least one attendant so that they had someone to care for their babies while they were being interviewed. RDW were interviewed using a structured questionnaire which was administered within 6 h of normal delivery.

#### Variables and measurements

A wealth quintile was considered based on possession of electronic items or vehicles [19]. The education level of women and their husbands was also taken into consideration. One of the inclusion criteria for the study was to enroll mothers who had at least one other baby or a history of delivery. The seven major danger signs used in this study were those outlined in the National health strategy of Bangladesh and World Health Organization’s (WHO) pocket book of inpatient newborn care [20]. A detailed description of the data collection tool and procedure are given in the Additional file 1.

#### Statistical analysis

A frequency distribution was used to present the categorical variables. Mean and standard deviation (SD) were used for continuous variables. This study used logistic regression to identify the association between husband involvement and knowledge of at least one danger sign among the RDW. Stata version 13 (College Station, Texas, USA) was used for data analysis. Crude odds ratio (CORs) and adjusted odds ratios (AORs) were calculated, and odds ratios were reported with 95% confidence intervals.

### Results

#### Participant’s characteristics

The mean age of the participants was 23.7 (± 8.6) years. More than half of the participants were residents of semi-urban areas. Around 64.8% of the participants had received a secondary education, and 62.3% were from households categorized as having a ‘low’ asset ownership. The majority of the interviewees were housewives (71.1%), and around 57.1% of all women had a previous history of hospital delivery (Table 1). Knowledge of ‘hyperthermia (i.e., fever)’ was the most well recognized danger sign, being recognized by 43.7% of the participants. Around 35% of the respondents were able to recall the danger signs of ‘hypothermia’, ‘lethargic’, ‘convulsions’, ‘severe chest indrawing’ and ‘stopped feeding well’ (see Additional file 2).

**Table 1:** Basic characteristics of study participants

Characteristics	Number	Percentage (%)
Maternal age, in years		
< 20	32	22.5
20–24	52	36.6
25–29	39	27.5
> 30	19	13.4
Mean age (SD)	23.7 (± 8.6)	
Place of residence		
Rural	59	41.6
Semi-urban	83	58.4
Occupation		
Housewife	101	71.1
Employed	41	28.9
Education level		
< Secondary	50	35.2
≥ Secondary	92	64.8
Number of children		
< 3	98	69.0
≥ 3	44	31.0
Number of ANC visit		
0	13	9.2
One visit	39	27.5
2–3 visits	58	40.8
≥ 4 visits	32	22.5
Husband’s education		
< Higher secondary	91	64.1
≥ Higher secondary	51	35.9
Household assets		
Low	87	61.3
High	55	38.7
Previous history of hospital delivery		
Yes	86	60.6
No	56	39.4
Husband’s involvement		
Yes	81	57.1
No	61	42.9

*ANC* antenatal care, *secondary* 10 class/grade, *higher secondary* 12 class

#### Factors influencing husband’s involvement in facility delivery

As shown in the bivariate logistic regression analysis in Table 2, factors that were significantly associated with the husband’s participation in the facility delivery (secondary outcome variable) included: increased age (COR: 1.4, 95% CI 1.2–2.1), semi-urban residence (COR: 1.3, 95% CI 1.1–1.5), secondary education (COR: 1.1, 95% CI 1.0–1.3), use of ANC services (COR: 1.2, 95% CI 1.1–1.3), and previous history of facility delivery (COR: 1.5, 95% CI 1.2–1.6).

**Table 2:** Factors associated with husband’s active involvement in facility delivery

Characteristics	Husband’s involved (n = 81)	COR (95% CI)	p value
Age, in years			
< 25	38 (46.9)	Ref.	–
≥ 25	43 (53.1)	1.4 (1.2–2.1)*	0.001
Location of residence			
Rural	32 (39.5)	Ref.	–
Semi-urban	49 (60.5)	1.3 (1.1–1.5)*	0.001
Occupation			
Housewife	56 (69.1)	Ref.	–
Employed	25 (30.9)	0.8 (0.6–1.1)	0.21
Education level			
< Secondary	33 (40.7)	Ref.	–
≥ Secondary	48 (59.3)	1.1 (1.0–1.3)*	0.001
Number of children			
< 3	56 (69.1)	Ref.	–
≥ 3	25 (30.9)	0.8 (0.7–1.1)	0.1
Number of ANC visit			
< 2 visits	35 (43.2)	Ref.	–
≥ 2 visits	46 (56.8)	1.2 (1.1–1.3)*	0.002
Husband’s education			
< Higher secondary	52 (64.2)	Ref.	–
≥ Higher secondary	29 (35.8)	0.9 (0.7–1.1)	0.41
Household assets			
Low	49 (60.5)	Ref.	–
High	32 (39.5)	0.9 (0.7–1.1)	0.32
Previous experience of facility delivery			
No	29 (35.8)	Ref.	–
Yes	52 (64.2)	1.5 (1.2–1.6)*	0.01

*ANC* antenatal care, *secondary* 10 class/grade, *higher secondary* 12 class, *ref.* reference value, *COR* crude odds ratio

* p < 0.05

Table 2 represents the bi-variable logistic regression only and we did not consider the multivariable analysis. We explored the different factors associated with the husband’s active involvement in taking the decision of facility delivery.

#### Factors associated with knowledge of newborn danger sign among the RDW

Table 3 describes the factors that were related to the outcome variable (i.e., knowledge of at least one danger sign) of this study. We found associations with secondary education (COR: 1.3, 95% CI 1.1–1.6), increased ANC visits (COR: 1.2, 95% CI 1.1–1.3), previous history of facility delivery (COR: 1.3, 95% CI 1.1–1.4), and husbands involvement in care (COR: 1.3, 95% CI 1.1–1.5) with having the maternal knowledge of at least one neonatal danger sign (Table 3). After adjusting for the maternal factors (high parity, ANC attendance, location of residence, occupation, household assets, facility-based delivery and education level), husband involvement (AOR: 1.2, 95% CI 1.1–1.4) was associated with an increased maternal knowledge of newborn danger signs.

**Table 3:** Factors associated with knowing at least one danger sign among the RDW

Characteristics	Danger sign identified (n = 73)	COR (95% CI)	A^a^OR (95% CI)
Age (years)			
< 25	38 (52.1)	Ref.	Ref.
≥ 25	35 (47.9)	0.8 (0.6–1.1)	0.9 (0.8–1.3)
Location of residence			
Rural	28 (38.4)	Ref.	Ref.
Semi-urban	45 (61.6)	1.1 (0.8–1.3)	0.9 (0.7–1.2)
Occupation			
Housewife	48 (65.8)	Ref.	Ref.
Employed	25 (34.2)	0.9 (0.6–1.1)	0.7 (0.5–1.0)
Education level			
< Secondary	21 (28.8)	Ref.	Ref.
≥ Secondary	52 (71.2)	1.3 (1.1–1.6)*	1.2 (1.1–1.5)*
Number of children			
< 3	52 (71.2)	Ref.	Ref.
≥ 3	21 (28.8)	0.9 (0.9–1.2)	0.8 (0.7–1.1)
ANC taken			
< 2 visits	22 (30.1)	Ref.	Ref.
≥ 2 visits	51 (69.9)	1.2 (1.1–1.3)*	1.1 (0.9–1.3)
Household assets			
Low	39 (53.4)	Ref.	Ref.
High	34 (46.6)	0.9 (0.8–1.1)	0.8 (0.7–1.1)
Husband’s involvement			
No	22 (30.1)	Ref.	Ref.
Yes	51 (69.9)	1.3 (1.1–1.5)*	1.2 (1.1–1.4)*
Previous experience of facility delivery			
No	21 (28.8)	Ref.	ref
Yes	52 (71.2)	1.2 (1.1–1.4)*	1.3 (1.1–1.4)*

*COR* crude odds ratio, *AOR* adjusted odds ratio, *ref.* reference value

* p < 0.05

^a^Adjusted for age, parity, ANC, residence, occupation, education, household assets, and facility delivery

### Discussion

This study found a significant association between husband involvement and maternal knowledge of newborn danger signs. Location of residence, increased maternal age, maternal education and previous history of facility delivery were also found to be associated with increased knowledge of mother in identifying the newborn danger signs. We also found that pregnant women attending two or more ANC appointments demonstrated an increased recall of neonatal danger signs, and husband involvement in these visits was associated with an increased ability to identify the danger signs. A previous study found that ANC visits encourage active birth pre-planning and preparedness [21]. During the antenatal check-up, pregnant women also get the opportunity to learn more about the maternal and newborn danger signs from healthcare professionals [22]. This could explain why increased access to ANC services tends to result in pregnant woman having greater knowledge about neonatal danger signs. It could be argued that mothers who have attended ANC services become motivated to access hospital-based delivery services. Consistent with previous studies in low to middle-income settings, we also found that high levels of education were positively associated with being able to recall at least one danger sign [23–25]. This is thought to be due to the higher ability of educated women to be able to understand messages related to health education [23]. Our study found that husband involvement was significantly associated with increased maternal knowledge of neonatal danger signs. This finding is consistent with the systematic review and meta-analysis conducted by Yargawa et al. [26], which showed that male or husband involvement in care was significantly associated with positive maternal outcomes [27, 28].

This study found that the correct response to ‘lethargic’ and ‘stopped feeding well’ was given by approximately 38% of the participants. Of the seven danger signs, the least known were ‘hypothermia,’ and ‘fast breathing’. In a study conducted in Pakistan, Khadduri et al. [29] had also identified ‘difficulty feeding’ and ‘fast breathing’ as the least known danger signs amongst a group of new mothers. This low knowledge of hypothermia is cause for concern considering it is a life-threatening condition for newborns. The delay in identification of these lesser known danger signs may be contributing to neonatal mortality and morbidity in low resource settings like Bangladesh [30]. In our study, knowledge on hyperthermia (fever) was found to be the most commonly known danger sign, and the sign was mentioned by 43.7% of the participants. Our findings are similar to other studies conducted in India which found here was a high awareness (75–90%) of ‘hyperthermia’ as a danger sign [31, 32].

More than half of the respondents (RDW) of our study were able to identify at least one key danger sign, and the findings were generally consistent with the results of other low-income settings [33]. The imperfect knowledge of newborn danger signs is one of the leading causes of delay in decision making and care-seeking that is also known as the first delay [34]. Waiswa et al. [35] argue that the first delay is the underlying cause of 50% of all neonatal deaths. However, danger sign identification and recognition by the mother might not always be enough to empower women to decide to access hospital or facility care for their babies in rural settings [10, 36]. The decision to seek care from a hospital for sick neonates may also be sought from elder family members depending on family dynamics. In addition to decision making, previous research identified major constraints like high cost, low socioeconomic status, difficulty in accessing to healthcare services and low literacy levels that may prevent a mother from taking her baby to the hospital [37].

This study identified increased age, semi-urban residence, secondary education, higher use of ANC services and previous history of facility delivery as factors associated with husband involvement in facility delivery. Husband involvement in pregnancy and reproductive health components has long been discussed. Some studies have shown benefits of involving men in reproductive health issues by emphasizing increased utilization of antenatal care and postnatal care that helps a mother in alleviating the stress, pain, and anxiety related to pregnancy and post-partum depression [27, 38–48]. These positive findings can be further explored by conducting in-depth qualitative studies. Moreover, husbands can improve their wives knowledge on self-care and newborn care by providing counseling, health education, and by advising them to practice increased health care seeking behaviour [49]. Our study also revealed that new mothers who got little input from their partners during pregnancy and delivery may have a poorer understanding of neonatal danger signs in comparison to women whose husbands are actively involved.

Finally, the results of our study highlight the vital role of the husband in optimizing the level of knowledge and awareness of the mother with regards to neonatal danger signs. Further longitudinal studies would be required to derive reliable inferences on the husband’s supportive role in the neonatal care-seeking paradigm by the mother and how it precludes neonatal mortality as a result of early identification of danger signs.

## Limitations

The results of this study were based on three sub-district hospitals. Although the participants of this study were roughly representative of the rural population of Bangladesh, the findings may not adequately reflect the actual situation given our sample size. Our sample size was relatively small; however, post hoc calculation determined a high statistical power, which is one of the strengths of this study. Also, the temporality of the association was not established given the cross-sectional design of the study. Despite adjustments for age, location of residence, occupation, education level, parity, household assets, ANC visits and previous facility delivery, there may be some unidentified confounding variables which were not taken into account. This study also did not consider whether the husband’s involvement in maternal care affects the mothers knowledge when mothers are better educated or when they have been trained to recognize the danger signs of neonatal illness.

## Additional files

**Additional file 1.** Data collection tool and procedure.

**Additional file 2.** Participants correctly identified the neonatal danger signs.

## Abbreviations

ANC: antenatal care
AOR: adjusted odds ratio
COR: crude odds ratio
NMR: neonatal mortality rate
RDW: Recently Delivered Women
SD: standard deviation
UHC: Upazila Health Complex
WHO: World Health Organization
