# Supplementary material for: Husband’s involvement with mother’s awareness and knowledge of newborn danger signs in facility-based childbirth settings: a cross-sectional study from rural Bangladesh
Source: BMC Res Notes. 2018 May 9;11:286. doi: 10.1186/s13104-018-3386-6 (PMC5944176; doi:10.1186/s13104-018-3386-6)
Supplement: Supplementary file 1 — Additional file 1. Data collection tool and procedure. [file 13104_2018_3386_MOESM1_ESM.docx]

**Additional file 1: Data collection tool and procedure**

| **Data collection tool**  A structured pre-tested questionnaire verified and finalized by a group of public health experts was used for collecting the data. The questionnaire was translated into the local language (Bengali) and afterward, it was translated back to English to check the translation consistency. Two study nurses identified the participants based on inclusion criteria in each facility. Six research assistants collected the data from the hospitals by face-to-face interview. Two medical officers supervised the overall data collection, and 5% of double data entry was performed to check the data quality. A technical team did quality control of the data entry process. The inclusion criteria were as follow:Mother willing to enroll into the studyPrevious history of child birth (at least one alive baby)Baby was delivered in hospital and the condition of mother was good (recent pregnancy outcome)Exclusion criteria:Mother with stillbirth or adverse fetal outcomeVery Low Birth Weight baby and unstable (need immediate referral)Mother who developed Post-Partum Haemorrhage (PPH)Variables and measurements Pregnancy and delivery related information  Age was categorized into ‘<20 years’, ‘20-25’, ‘26-30’and ‘>30 years’ groups. Occupation was classified as housewife and employed. The group of women who took care of the family and did the household activities were termed as a housewife in this study, and employed were those women who worked outside the home and earned a livelihood for her family. A wealth quintile was considered to assess the socio-economic condition based on the possession of electronic items, devices or vehicles [19]. The participants were asked whether they possessed radio, mobile phone, television, computer, bicycle, motorcycle and CD player. If one participant possessed less than 2 items, it was considered as low asset category. Consequently, rest of the participants were categorized as moderate (any three). The education level of women and their husbands were taken into consideration to include in this study. For the RDW, education status was categorized as <SSC (10 class/grade) and ≥SSC (10 class) according to Bangladesh education system. However, we considered <HSC (12 class/grade) and ≥HSC (12 grade) cut off point for the husband education status. RDW was asked about the number of antenatal visits they received during pregnancy. They were also asked about the parity and history of previous delivery whether RDW took facility based previous delivery at UHC.  Outcome variable (Knowledge on newborn danger sign)  RWD was asked about danger signs of neonates. The question was asked, “Can you please tell me what the danger sign of neonates are?” Spontaneous responses were categorized according to the group. The major seven danger signs were incorporated from the National health strategy of Bangladesh and World Health Organization’s (WHO) pocketbook of inpatient newborn care [20]. These signs were: hypothermia, hyperthermia, convulsion, lethargic, fast breathing, stopped feeding well and severe chest in-drawing. They were also asked whether they considered these danger signs to bring her child to the hospital for seeking care.  Husband’s involvement  The interviewers also asked the RDW whether their husband accompanied them to come facility for delivery. Husband’s involvement was defined by the presence of husband’s involvement during antenatal, delivery and post-natal care. This study also looked at degree of the husband’s participation, level of support that was made about his wife’s delivery.  Husband’s involvement was defined by the inclusion of all the following criteria:   1. Presence of husband at the time of delivery 2. Husband’s took appropriate care during the pregnancy period of wife 3. Husband accompanied his wife to take at least one facility based ANC 4. Gave financial support to RDW for birth preparedness   Pregnancy and delivery related information  The interviewers also asked the RDW whether they were accompanied by their husband when they came to delivery. They were asked whether they would come for PNC care to the hospital if the baby gets sick. They RDW were also wished to know whether they took the birth preparation during coming to the hospital. The term birth preparedness included activities such as buying and bringing warm clothes for baby, some money, foods and arranging the transportation and an accompanied person to help her. |
| --- |

RDW: Recently Delivered Women; ANC: Antenatal Care; PNC: Postnatal Care; SSC: Secondary School Certificate (10 class/grade); HSC: Higher Secondary Certificate (12 class/grade); UHC: Upazila Health Complex (sub-district hospital)
