# Supplementary material for: Husband’s involvement with mother’s awareness and knowledge of newborn danger signs in facility-based childbirth settings: a cross-sectional study from rural Bangladesh
Source: BMC Res Notes. 2018 May 9;11:286. doi: 10.1186/s13104-018-3386-6 (PMC5944176; doi:10.1186/s13104-018-3386-6)
Supplement: Supplementary file 2 — Additional file 2. Participants correctly identified the neonatal danger signs. [file 13104_2018_3386_MOESM2_ESM.docx]

**Additional file 2: Participants correctly identified the neonatal danger signs**

| **Knowledge items**  (Neonatal danger signs) | | **Participants who correctly identified**  n (%) | **Husband involved group**  n (%) |
| --- | --- | --- | --- |
| 1 | Fast breathing | 47 (33.0) | 30 (63.8) |
| 2 | Lethargic | 56 (39.4) | 35 (62.5) |
| 3 | Convulsions | 48 (33.8) | 30 (62.8) |
| 4 | Hypothermia | 37 (26.1) | 26 (68.9) |
| 5 | Stopped feeding well | 54 (38.0) | 38 (70.4) |
| 6 | Severe chest in-drawing | 46 (32.4) | 29 (63.2) |
| 7 | Hyperthermia | 62 (43.7) | 44 (70.6) |

n (%): number (percentage) of participants
